# Pre-exposure prophylaxis (PrEP) among people who use drugs: a qualitative scoping review of implementation determinants and change methods

**DOI:** 10.1186/s13722-024-00478-2

**Published:** 2024-05-30

**Authors:** James L. Merle, Juan P. Zapata, Artur Quieroz, Alithia Zamantakis, Olutobi Sanuade, Brian Mustanski, Justin D. Smith

**Affiliations:** 1 Department of Population Health Sciences, Spencer Fox Eccles School of Medicine, University of Utah, Salt Lake City, UT USA; 2 Department of Psychiatry and Behavioral Sciences, Northwestern University, Chicago, IL USA; 3 Institute for Sexual and Gender Minority Health and Wellbeing, Northwestern University, Chicago, IL USA; 4 Medical Social Sciences Department, Northwestern University, Chicago, IL USA; 5 Department of Infectious Diseases, Northwestern University, Chicago, IL USA

**Keywords:** HIV, Pre-exposure prophylaxis, People who use drugs, Implementation science, Determinants, Barriers, Facilitators, Implementation strategies, Adjunctive interventions, Consolidated Framework for Implementation Research

## Abstract

Implementation of pre-exposure prophylaxis (PrEP) to prevent HIV transmission is suboptimal in the United States, particularly among people who use drugs (PWUD). PrEP research among PWUD is scarce, and the factors that impact implementation are largely unknown. Therefore, we conducted a scoping review of implementation determinants (i.e., barriers and facilitators), as well as the change methods (implementation strategies and adjunctive interventions) that have been evaluated to increase PrEP implementation and use among PWUD. We identified 32 peer-reviewed articles assessing determinants and five that evaluated change methods. Determinants were coded using the updated Consolidated Framework for Implementation Research (CFIR), which is an established framework to understand the multilevel barriers and facilitators associated with implementation. Findings indicate that most research was conducted among PrEP recipients (i.e., patients), focusing on awareness and willingness to use PrEP, with less focus on factors impacting clinicians and service delivery systems. Moreover, very few change methods have been evaluated to improve clinician adoption and adherence to CDC guidelines for PrEP provision and/or recipient uptake and adherence to PrEP. Future research is needed that focuses on factors impacting implementation from a clinician standpoint as well as innovative change methods to increase PrEP awareness, reach, adoption, and sustained adherence to guidelines. Implementation Science offers a wealth of knowledge to speed up the effort to end the HIV epidemic in the United States.

**Supplementary Information:**

The online version contains supplementary material available at 10.1186/s13722-024-00478-2.

## Introduction

HIV transmission among people who use drugs (PWUD) is an ongoing public health concern in the U.S [1]. The association between drug use and HIV transmission is well-established; PWUD face a high risk of acquiring HIV through injection and sexual exposures [2, 3]. The underlying factors contributing to HIV vulnerability among PWUD are multifaceted. The use of stimulants such as methamphetamine, which is increasingly prevalent among social networks of cisgender men who have sex with men (MSM) [4], is associated with heightened impulsivity, behavioral disinhibition, engagement in condomless anal or vaginal sex, and multiple sexual partnerships [5]. Numerous studies have also consistently shown that PWUD are more prone to engaging in behaviors that can transmit HIV, such as having condomless sex and sharing syringes for drug injection [6, 7]. In fact, data from the U.S National Behavioral Surveillance Survey reveals that approximately three-quarters of sampled people who inject drugs (PWID) reported engaging in receptive syringe sharing and/or condomless sex in the past year [8].

The injection of opioids, such as heroin, along with the presence of illicitly manufactured fentanyl in local drug supplies has been linked to subsequent HIV outbreaks [9, 10]. Despite efforts to address illicit drug use, the use of methamphetamine via injection and other routes of administration has been on the rise [11]. These outbreaks are hindering progress in combating the HIV epidemic, as they are closely tied to increased opioid and polysubstance use [12]. Although effective biomedical prevention technologies like pre-exposure prophylaxis (PrEP) have been introduced for PWUD at high risk of HIV infection [13], the implementation of PrEP among PWUD remains limited despite their suitability as candidates for PrEP and the expressed willingness of PWUD to take PrEP [14, 15]. Consequently, implementing prevention interventions such as PrEP among PWUD can mitigate drug-related HIV transmissions [16].

PrEP, currently available in the form of an oral antiretroviral regimen or a bimonthly injection, has proven to be highly effective in reducing HIV acquisition through sexual exposure [17, 18]. Clinical trials conducted in diverse settings, including studies involving cisgender MSM, transgender women, heterosexual couples, and PWID, have demonstrated a reduction in HIV acquisition ranging from 44 to 75% [19]. PrEP also provides protection from HIV via injection drug use, though it has been shown to be less effective among PWID [18]. We identified only one study that enrolled PWID, and this study was associated with a 49% percent reduction in HIV risk—toward the bottom of the range of effectiveness. This study also took place in Bangkok, where needle exchange was not available, adherence was low, and researchers were not able to differentiate sexually from parenterally acquired HIV [20]. Moreover, optimal adherence to PrEP has shown a significant increase in efficacy, with rates ranging from 80 to 99% [20, 21]. Although PrEP usage data among PWID is scarce, studies indicate that limited utilization and poor adherence are driven by socioecological and structural factors [22]. Differences have also been found by age and gender, injection patterns, types of drugs used, as well as experiences of incarceration and homelessness [23]. However, there is an overall dearth of research seeking to understand and overcome these barriers, leading to a limited comprehension of the determinants that influence PrEP implementation among PWID, and PWUD more generally.

## Determinants of PrEP implementation

Implementation science offers valuable insights into bridging the gap between scientific evidence and the utilization of PrEP [24, 25]. The effective implementation of evidence-based interventions is influenced by contextual factors, including barriers and facilitators. These determinants, which are essential for deploying, modifying, enhancing, or discontinuing evidence-based interventions, play a crucial role [26]. Although several frameworks exist to organize and identify these determinants, the Consolidated Framework for Implementation Research (CFIR) uses a comprehensive approach for understanding and addressing the multifaceted aspects of implementation. The updated CFIR [27] comprises five main domains: *Outer Setting*, *Inner Setting*, *Innovation*, *Process*, and *Individuals*. Each domain includes multiple constructs, which are detailed in the Methods section.

### Change methods for PrEP

Implementation strategies encompass the methods and techniques used to enhance implementation outcomes in evidence-based programs or practices by addressing barriers and leveraging facilitators [28]. Implementation outcomes are distinct from service or client outcomes and primarily focus on the adoption, fidelity, penetration, and sustainability of these programs by implementers and implementing delivery systems [29]. In the context of the PrEP care continuum [30], implementation strategies play a critical role in increasing awareness and adoption among PrEP providers, including healthcare professionals and pharmacists. These strategies aim to strengthen the knowledge, skills, and resources necessary for identifying individuals at risk and prescribing PrEP, while also facilitating access for eligible patients [31–33].

In contrast to implementation strategies that focus on the delivery system, adjunctive interventions aim to address determinants among intervention recipients or end-users [34, 35]. Adjunctive interventions serve as supplementary methods to enhance the effectiveness of clinical interventions like PrEP, by targeting recipient uptake and adherence. Examples of adjunctive interventions for PrEP include digital tools that provide reminders, peer support programs, and individual adherence counseling [36–39]. These interventions are designed to assist recipients in initiating and maintaining adherence to PrEP. We refer to implementation strategies and adjunctive interventions collectively as change methods.

### Existing literature

Numerous studies have extensively examined PrEP awareness, knowledge, willingness to use, PrEP utilization, and adherence among PWUD. The majority of studies have primarily focused on socio-demographic barriers encountered by PWUD in accessing PrEP, including factors such as age, gender, financial constraints, and limited healthcare engagement [40–43]. For example, Gebru et al. [40] conducted a systematic review on the measurement of substance use and PrEP adherence in studies involving MSM and transgender women. Their findings revealed mixed results regarding the association between substance use and PrEP adherence. Notably, alcohol use showed the strongest correlation with poorer PrEP adherence. In a review focusing on cisgender female sex workers and/or women who use drugs (WWUD) and their engagement in the PrEP care continuum, Glick et al. [43] identified that studies consistently reported a significant lack of PrEP awareness, however, willingness to take PrEP was higher once those individuals knew it existed. Authors also indicated a dearth of research examining PrEP uptake and adherence for these populations. Similarly, in a meta-analytic review examining PrEP awareness and willingness to use among WWUD in the U.S., 21% were aware, and after being informed about PrEP, willingness to use PrEP increased to 60% among both injectors and non-injectors, and 57% among injectors alone [44].

To address the disparities in PrEP uptake and access among PWUD, it is crucial to employ targeted change methods, including implementation strategies and adjunctive interventions. However, determining the most effective methods for this specific population remains unclear. Equally understudied are the implementation determinants and strategies to improve system and provider implementation of PrEP, thereby increasing access and reach among PWUD. Notably, no previous review has examined implementation determinants using the updated CFIR framework, which includes greater attention to intervention recipients than the original CFIR (among other changes), and no study has adequately reviewed the available change methods to enhance PrEP implementation within service settings tailored for PWUD populations. To contribute to the existing literature, we conducted a scoping review and catalogued implementation determinants using the updated CFIR across all PWUD populations. Additionally, we outline change methods for PrEP, focusing specifically on PWUD, building upon a larger systematic review (Li et al., 2022) of PrEP implementation determinants for all populations. This review aims to answer the following research questions:

1. What barriers and facilitators have been identified as determinants of PrEP implementation for PWUD?
2. What change methods have been implemented to address PrEP implementation determinants in settings serving PWUD?
3. What gaps in the literature can be identified to improve implementation outcomes for PWUD and the settings that serve this population?

### Methods

**Fig. 1:**
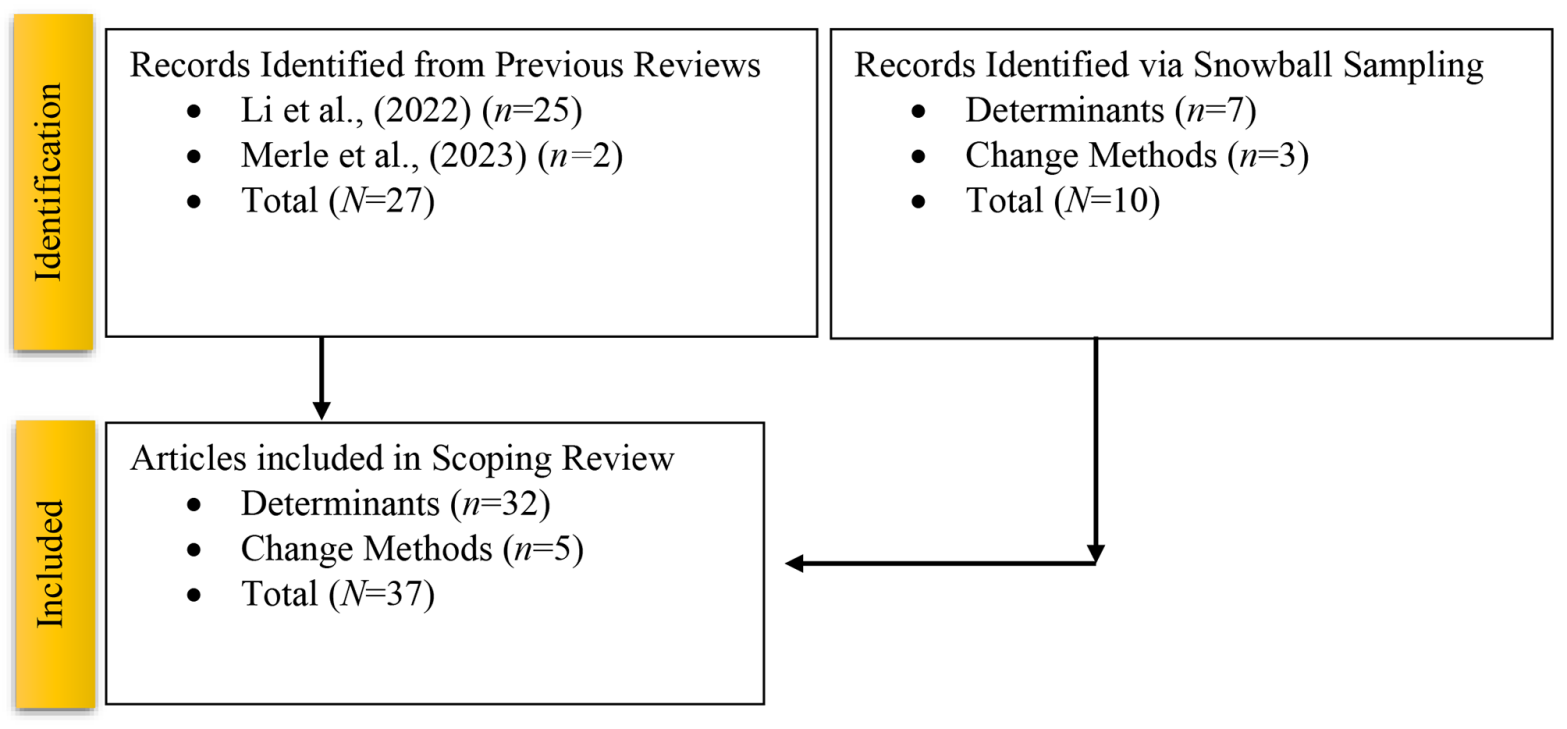
PRISMA-ScR flowchart for PrEP implementation among people who use drugs

We conducted a scoping review to examine PrEP determinants and change methods among PWUD within two previously published systematic reviews: (a) PrEP determinants [45], which identified 353 articles, and (b) PrEP change methods [35] which identified 44 articles. Full search details have also been reported elsewhere [46]. In this scoping review, we also used snowball sampling to identify additional articles not included in these reviews as well as those published since. Figure 1 displays the PRISMA-ScR. All included studies are displayed in Table 1.

**Table 1:** Included study references

**Determinants**	
Adams LM, Balderson BH. HIV providers’ likelihood to prescribe pre-exposure prophylaxis (PrEP) for HIV prevention differs by patient type: a short report. AIDS Care. 2016;28(9):1154-8; 10.1080/09540121.2016.1153595.	
Biello KB, Bazzi AR, Mimiaga MJ, Biancarelli DL, Edeza A, Salhaney P, et al. Perspectives on HIV pre-exposure prophylaxis (PrEP) utilization and related intervention needs among people who inject drugs. Harm Reduction Journal. 2018;15(1):55; 10.1186/s12954-018-0263-5.	
Calabrese SK, Tekeste M, Mayer KH, Magnus M, Krakower DS, Kershaw TS, et al. Considering Stigma in the Provision of HIV Pre-Exposure Prophylaxis: Reflections from Current Prescribers. AIDS Patient Care STDS. 2019;33(2):79–88; 10.1089/apc.2018.0166.	
Chan PA, Mena L, Patel R, Oldenburg CE, Beauchamps L, Perez-Brumer AG, et al. Retention in care outcomes for HIV pre-exposure prophylaxis implementation programmes among men who have sex with men in three US cities. J Int AIDS Soc. 2016;19(1); 10.7448/IAS.19.1.20903.	
Chan PA, Patel RR, Mena L, Marshall BD, Rose J, Sutten Coats C, et al. Long-term retention in pre-exposure prophylaxis care among men who have sex with men and transgender women in the United States. J Int AIDS Soc. 2019;22(8):e25385; 10.1002/jia2.25385.	
Edelman EJ, Moore BA, Calabrese SK, Berkenblit G, Cunningham C, Patel V, et al. Primary Care Physicians’ Willingness to Prescribe HIV Pre-exposure Prophylaxis for People who Inject Drugs. AIDS Behav. 2017;21(4):1025-33; 10.1007/s10461-016-1612-6.	
Felsher M, Ziegler E, Amico KR, Carrico A, Coleman J, Roth AM. “PrEP just isn’t my priority”: Adherence challenges among women who inject drugs participating in a pre-exposure prophylaxis (PrEP) demonstration project in Philadelphia, PA USA. Soc Sci Med. 2021;275:113809; 10.1016/j.socscimed.2021.113809.	
Felsher M, Ziegler E, Smith LR, Sherman SG, Amico KR, Fox R, et al. An Exploration of Pre-exposure Prophylaxis (PrEP) Initiation Among Women Who Inject Drugs. Arch Sex Behav. 2020;49(6):2205-12; 10.1007/s10508-020-01684-0.	
Grov C, Rendina HJ, John SA, Parsons JT. Determining the Roles that Club Drugs, Marijuana, and Heavy Drinking Play in PrEP Medication Adherence Among Gay and Bisexual Men: Implications for Treatment and Research. AIDS Behav. 2019;23(5):1277-86; 10.1007/s10461-018-2309-9.	
Horack CL, Newton SL, Vos M, Wolfe BA, Whitaker A. Pre-Exposure Prophylaxis in a Reproductive Health Setting: A Quality Improvement Project. Health Promot Pract. 2020;21(5):687-9; 10.1177/1524839920923275.	
Krakower DS, Beekmann SE, Polgreen PM, Mayer KH. Diffusion of Newer HIV Prevention Innovations: Variable Practices of Frontline Infectious Diseases Physicians. Clin Infect Dis. 2016;62(1):99–105; 10.1093/cid/civ736.	
Krakower DS, Ware NC, Maloney KM, Wilson IB, Wong JB, Mayer KH. Differing Experiences with Pre-Exposure Prophylaxis in Boston Among Lesbian, Gay, Bisexual, and Transgender Specialists and Generalists in Primary Care: Implications for Scale-Up. AIDS Patient Care STDS. 2017;31(7):297–304; 10.1089/apc.2017.0031.	
Kuo I, Olsen H, Patrick R, Phillips G, 2nd, Magnus M, Opoku J, et al. Willingness to use HIV pre-exposure prophylaxis among community-recruited, older people who inject drugs in Washington, DC. Drug Alcohol Depend. 2016;164:8–13; 10.1016/j.drugalcdep.2016.02.044.	
Mimiaga MJ, Case P, Johnson CV, Safren SA, Mayer KH. Preexposure antiretroviral prophylaxis attitudes in high-risk Boston area men who report having sex with men: limited knowledge and experience but potential for increased utilization after education. J Acquir Immune Defic Syndr. 2009;50(1):77–83; 10.1097/QAI.0b013e31818d5a27.	
Mimiaga MJ, Closson EF, Kothary V, Mitty JA. Sexual partnerships and considerations for HIV antiretroviral pre-exposure prophylaxis utilization among high-risk substance using men who have sex with men. Arch Sex Behav. 2014;43(1):99–106; 10.1007/s10508-013-0208-8.	
Oldenburg CE, Mitty JA, Biello KB, Closson EF, Safren SA, Mayer KH, et al. Differences in Attitudes About HIV Pre-Exposure Prophylaxis Use Among Stimulant Versus Alcohol Using Men Who Have Sex with Men. AIDS Behav. 2016;20(7):1451-60; 10.1007/s10461-015-1226-4.	
Peitzmeier SM, Tomko C, Wingo E, Sawyer A, Sherman SG, Glass N, et al. Acceptability of microbicidal vaginal rings and oral pre-exposure prophylaxis for HIV prevention among female sex workers in a high-prevalence US city. AIDS Care. 2017;29(11):1453-7; 10.1080/09540121.2017.1300628.	
Qin Y, Price C, Rutledge R, Puglisi L, Madden LM, Meyer JP. Women’s Decision-Making about PrEP for HIV Prevention in Drug Treatment Contexts. J Int Assoc Provid AIDS Care. 2020;19:2325958219900091; 10.1177/2325958219900091.	
Roth AM, Aumaier BL, Felsher MA, Welles SL, Martinez-Donate AP, Chavis M, et al. An Exploration of Factors Impacting Preexposure Prophylaxis Eligibility and Access Among Syringe Exchange Users. Sex Transm Dis. 2018;45(4):217 − 21; 10.1097/olq.0000000000000728.	
Rutledge R, Madden L, Ogbuagu O, Meyer JP. HIV Risk perception and eligibility for pre-exposure prophylaxis in women involved in the criminal justice system. AIDS Care. 2018;30(10):1282-9; 10.1080/09540121.2018.1447079.	
Shrestha R, Altice F, Karki P, Copenhaver M. Developing an Integrated, Brief Biobehavioral HIV Prevention Intervention for High-Risk Drug Users in Treatment: The Process and Outcome of Formative Research. Front Immunol. 2017;8:561; 10.3389/fimmu.2017.00561.	
Shrestha R, Altice FL, Huedo-Medina TB, Karki P, Copenhaver M. Willingness to Use Pre-Exposure Prophylaxis (PrEP): An Empirical Test of the Information-Motivation-Behavioral Skills (IMB) Model among High-Risk Drug Users in Treatment. AIDS Behav. 2017;21(5):1299 − 308; 10.1007/s10461-016-1650-0.	
Shrestha R, Copenhaver M. Exploring the Use of Pre-exposure Prophylaxis (PrEP) for HIV Prevention Among High-Risk People Who Use Drugs in Treatment. Front Public Health. 2018;6:195; 10.3389/fpubh.2018.00195.	
Shrestha R, Karki P, Altice FL, Dubov O, Fraenkel L, Huedo-Medina T, et al. Measuring Acceptability and Preferences for Implementation of Pre-Exposure Prophylaxis (PrEP) Using Conjoint Analysis: An Application to Primary HIV Prevention Among High Risk Drug Users. AIDS Behav. 2018;22(4):1228-38; 10.1007/s10461-017-1851-1.	
Shrestha R, Karki P, Altice FL, Huedo-Medina TB, Meyer JP, Madden L, et al. Correlates of willingness to initiate pre-exposure prophylaxis and anticipation of practicing safer drug- and sex-related behaviors among high-risk drug users on methadone treatment. Drug Alcohol Depend. 2017;173:107 − 16; 10.1016/j.drugalcdep.2016.12.023.	
Smith DK, Mendoza MC, Stryker JE, Rose CE. PrEP Awareness and Attitudes in a National Survey of Primary Care Clinicians in the United States, 2009–2015. PLoS One. 2016;11(6):e0156592; 10.1371/journal.pone.0156592.	
Spector AY, Remien RH, Tross S. PrEP in substance abuse treatment: a qualitative study of treatment provider perspectives. Subst Abuse Treat Prev Policy. 2015;10:1; 10.1186/1747-597x-10-1.	
Stein M, Thurmond P, Bailey G. Willingness to use HIV pre-exposure prophylaxis among opiate users. AIDS Behav. 2014;18(9):1694 − 700; 10.1007/s10461-014-0778-z.	
Teitelman AM, Chittamuru D, Koblin BA, Davis A, Brawner BM, Fiore D, et al. Beliefs Associated with Intention to Use PrEP Among Cisgender U.S. Women at Elevated HIV Risk. Arch Sex Behav. 2020;49(6):2213-21; 10.1007/s10508-020-01681-3.	
Towe SL, Sullivan CA, McKellar MS, Meade CS. Examining the Potential of Pre-exposure Prophylaxis (PrEP) for HIV Prevention in a Community Sample of Persons Who Use Stimulants Living in the Southern United States. AIDS Behav. 2021;25(5):1480-9; 10.1007/s10461-020-02987-y.	
Underhill K, Morrow KM, Colleran CM, Holcomb R, Operario D, Calabrese SK, et al. Access to healthcare, HIV/STI testing, and preferred pre-exposure prophylaxis providers among men who have sex with men and men who engage in street-based sex work in the US. PLoS One. 2014;9(11):e112425; 10.1371/journal.pone.0112425.	
Walters SM, Reilly KH, Neaigus A, Braunstein S. Awareness of pre-exposure prophylaxis (PrEP) among women who inject drugs in NYC: the importance of networks and syringe exchange programs for HIV prevention. Harm Reduct J. 2017;14(1):40; 10.1186/s12954-017-0166-x.	
**Change Methods**	
Bartholomew TS, Andraka-Cristou B, Totaram RK, Harris S, Doblecki-Lewis S, Ostrer L, et al. “We want everything in a one-stop shop”: acceptability and feasibility of PrEP and buprenorphine implementation with mobile syringe services for Black people who inject drugs. Harm Reduction Journal. 2022;19(1):133; 10.1186/s12954-022-00721-6.	
Meyer J, Price C, Tracey D, Sharpless L, Song Y, Madden L, et al. Preference for and Efficacy of a PrEP Decision Aid for Women with Substance Use Disorders. Patient Preference and Adherence. 2021;15:1913-27; 10.2147/PPA.S315543.	
Richterman A, Ghadimi F, Teitelman AM, Moore K, Acri T, North H, et al. Acceptability and Feasibility of a Mobile Phone Application to Support HIV Pre-exposure Prophylaxis Among Women with Opioid Use Disorder. AIDS and Behavior. 2023; 10.1007/s10461-023-04060-w.	
Roth AM, Tran NK, Felsher M, Gadegbeku AB, Piecara B, Fox R, et al. Integrating HIV Preexposure Prophylaxis With Community-Based Syringe Services for Women Who Inject Drugs: Results From the Project SHE Demonstration Study. J Acquir Immune Defic Syndr. 2021;86(3):e61-e70; 10.1097/qai.0000000000002558.	
Shrestha R, Altice FL, Karki P, Copenhaver MM. Integrated Bio-behavioral Approach to Improve Adherence to Pre-exposure Prophylaxis and Reduce HIV Risk in People Who Use Drugs: A Pilot Feasibility Study. AIDS and Behavior. 2018;22(8):2640-9; 10.1007/s10461-018-2099-0.	

### Study identification and inclusion criteria

We identified a subset of implementation determinant papers included in the review by Li et al. [45] using the HIV Implementation Literature Review Dashboard (https://hivimpsci.northwestern.edu/dashboard/), which was developed as a publicly accessible tool. We used the filter functions to identify articles that met the following criteria: (a) study population, participants, and settings are U.S.-based, (b), study focuses on intersecting PWUD priority populations (e.g., Black/African American PWUD), (c) study is related to PrEP, (d) the study contains original empirical research, (e) the study outcomes are related to dissemination and implementation science. This search yielded 25 articles from the dashboard. We also used snowball sampling methods by reviewing the citations of these articles to identify an additional seven articles. Together, 32 studies were coded using MAXQDA, a mixed-methods data analysis software [47].

### Determinant coding procedures

Each determinant was coded by valence (i.e., barrier vs. facilitator); the method of data collection (i.e., quantitatively, qualitatively, or by use of mixed or multi methods); the setting(s) which participants were identified; whether the sample included people who inject drugs intravenously or people who use drugs or substances non-intravenously; and if the type of drug or substance was detailed, this was also captured by categories based on drug effects [48].

Additionally, articles were coded using an adapted version of the updated Consolidated Framework for Implementation Research (CFIR) to identify the determinant and construct [27]. The CFIR is broken down into five domains: *Innovation* (i.e., factors related to PrEP being used or implemented), *Inner Setting* (i.e., the setting in which PrEP is implemented; e.g., hospital, community organization), *Outer Setting* (i.e., the social, political, cultural context in which deliver and uptake occur), *Process* (i.e., the activities and strategies needed to implement or use PrEP), and *Individuals* (i.e., roles and characteristics of individuals implementing or using PrEP). Finally, the Individuals domain is broken down into subdomains related to individual’s role (i.e., innovation deliverer [provider], clinician or innovation recipient [patient]) and characteristics associated with behavior change via the COM-B system [49]: namely, Capability, Opportunity, and Motivation associated with the individual’s behavior. Capability refers to an individual’s knowledge, memory, attention and decision processes, physical and psychological ability to carry out a behavior, as well as an ability to regulate one’s own behavior. Opportunity involves awareness, availability, scope, social influences and environmental context and resources. Motivation includes an individual’s reflective beliefs about their capabilities, beliefs about the consequences of enacting or changing behavior, their willingness, goals and intentions around enacting behavior, as well as their optimism and emotional responses.

We adapted the CFIR2.0 by expanding it to not only include *implementation* determinants, which capture delivery setting-level barriers and facilitators, but also *innovation* determinants, which capture recipient (i.e., patient-level) barriers and facilitators. Although not explicitly captured by CFIR2.0, These determinants are important because most of the literature related to PrEP focuses on patient-level determinants [45]. Therefore, we expanded our codebook to be able to capture patient-level perspectives at each CFIR domain level, as is similarly described in Li et al. [45].

Our determinant coding team consisted of five PhD level researchers with varying expertise in implementation science, HIV, and substance use. Each coder had prior experience using CFIR2.0 to code literature. The coding team met as a full group on two occasions for training and consensus on the first two articles. Then, 38% of the remaining articles (*k =* 12) were double-coded, with consensus meetings to ensure consistency. The remaining 19 articles were then coded individually by coders. The first author adjudicated any disagreements between coders and reviewed each code prior to analysis in consultation with the senior author. Our full determinant codebook with operational definitions for each code is provided as Supplemental File 1.

### Change method coding

We also identified two change methods papers from Merle et al. [35] and an additional three from snowball sampling (i.e., searching included article citations and utilizing Google Scholar to identify forward citations). For implementation strategies, we used the Expert Recommendations for Implementing Change (ERIC) taxonomy, which identifies 73 discrete implementation strategies categorized into 9 domains based on conceptual similarity to categorize each of the components within each strategy [50]. For adjunctive interventions, given that they involve different targets, outcomes, and change processes compared to implementation strategies (see Smith et al., 2024; esp. Table 1) [34], we coded the individual-level change processes within the core components of the interventions with the Theoretical Domains Framework (TDF) along with the Capability, Opportunity, Motivation, Behavior (COM-B) taxonomy [49, 51, 52]. To support our coding, we consulted with tools such as the theory and technique tool [53], which provides links between behavior change techniques and mechanisms of action. Our change method coding team consisted of three PhD level researchers with extensive prior experience using ERIC and the TDF (authors JLM, OS, JPZ). Each article was double coded and consensus on each code was achieved [46].

## Results

### Determinants

A total of 296 unique, measured determinants (N) were identified and analyzed across 32 studies (K). In terms of valence, most of these determinants (160, or 54%) were categorized as barriers, with 110 (37%) classified as facilitators. Additionally, 6 (2%) determinants were classified as both barriers and facilitators, 15 (5%) were categorized as neither, and 5 (2%) were unspecified or had uncertain valence. Regarding the method of data collection, qualitative methods were employed for most determinants (176, or 59%), while 120 (41%) were collected quantitatively.

### Delivery settings

Studies took place in a variety of settings. The most common setting was non-HIV specific clinical settings (primary care or general hospital setting, *k =* 10), followed by substance use treatment centers (including methadone programs, *k* = 7), community settings (including community-based organizations as well as community-wide surveys or surveillance data, and other community-based settings such as bars, *k* = 7), HIV-specific clinical settings(*k* = 3), syringe services programs (*k* = 2), those conducted in research offices (*k =* 2), and those conducted in the criminal justice system (*k =* 1).

### Mode and type of drug use

The majority of coded determinants were found among PWIDs (*n* = 126, 54%), with 32 (14%) classified as non-injecting (i.e., PWUD), and 74 (32%) representing a disaggregated sample of drug use methods. When examining type of drug or substance used, most were not specified by type (*n =* 152, 47%). Among reported drug type, stimulants were the most commonly reported (*n* = 74, 24%), followed by narcotic analgesics (*n* = 40, 13%), depressants (*n* = 24, 8%), hallucinogens (*n* = 12, 4%), dissociative anesthetics (*n* = 5, 2%), cannabis (*n* = 4, 1%), and inhalants (*n* = 2, < 1%). It is important to note that the total number (N) for mode and type does not equal 296, as some determinants were associated with multiple drugs. Additionally, for determinants related to providers, no specific drug or mode was identified.

### CFIR2.0 determinants

In this section, we present the results of CFIR2.0 coding. Table 2 provides a numerical breakdown of each CFIR2.0 domain and construct. Most determinants (*n =* 199, 67%) were classified as *innovation* determinants and 97 (33%) were *implementation* determinants. An overwhelming majority of determinants (*n* = 180, 57%) were described in the *Individuals* domain, pertaining to either patient or provider use or implementation of PrEP. *Outer Setting* had the second-highest number of determinants (*n* = 47, 15%), followed by (*n =* 43, 14%), *Inner Setting* (*n* = 23, 7%), and *Process* (*n =* 23, 7%). It should be noted that, on a few occasions, determinants were associated with more than one CFIR2.0 code; and at other times, determinants were not given a CFIR2.0 code due to lack of fit with the framework. Therefore, the total number of coded CFIR determinants (*N* = 316) is not equal to the number of determinants reported earlier in the results.

**Table 2:** Numerical breakdown of each CFIR domain and construct

CFIR 2.0 Domain & Construct		# of Coded Determinants	Proportion across all determinants(%)
**Innovation Characteristics**	Innovation source	2	0.6%
	Evidence-Base	6	1.9%
	Relative Advantage	2	0.6%
	Adaptability	1	0.3%
	Trialability	0	0.0%
	Complexity	5	1.6%
	Design	4	1.3%
	Cost	9	2.8%
	Other intervention characteristics (e.g., side effects)	14	4.4%
	**Subtotal**	43	13.6%
**Outer Setting**	Critical Incidents	3	0.9%
	Local Attitudes	14	4.4%
	Local Conditions	13	4.1%
	Partnerships & Connections	2	0.6%
	External Pressure	0	0.0%
	Policies & Laws	1	0.3%
	Financing	6	1.9%
	Structural/Systemic Oppression	8	2.5%
	**Subtotal**	47	14.9%
**Inner Setting**	Structural Characteristics	3	0.9%
	Physical Infrastructure	6	1.9%
	Information Technology Infrastructure	0	0.0%
	Work Infrastructure	1	0.3%
	Relational Connections	1	0.3%
	Communication	4	1.3%
	Culture	1	0.3%
	Equity-Centeredness	0	0.0%
	Recipient-Centeredness	2	0.6%
	Deliverer-Centeredness	0	0.0%
	Learning-Centeredness	0	0.0%
	Tension for Change	0	0.0%
	Compatibility	1	0.3%
	Relative Priority	0	0.0%
	Incentive Systems	0	0.0%
	Mission Alignment	0	0.0%
	Available Resources	0	0.0%
	Funding	1	0.3%
	Space	0	0.0%
	Materials & Equipment	0	0.0%
	Access to Knowledge & Information	1	0.3%
	Staffing	2	0.6%
	**Subtotal**	**23**	**7.2%**
**Characteristics of Individuals—Providers**	Capability	6	1.9%
	Opportunity	1	0.3%
	Motivation	15	4.7%
	Other individual characteristics not associated with behavior change	11	3.5%
	**Subtotal**	**33**	**10.4%**
**Characteristics of Individuals–Patients**	Capability	36	11.4%
	Opportunity	25	7.9%
	Motivation	45	14.2%
	Other individual characteristics not associated with behavior change	41	13.0%
	**Subtotal**	**147**	**46.5%**
**Process**	Teaming	0	0%
	Assessing for needs: Innovation Deliverers	1	0.3%
	Assessing for needs: Innovation Recipients	0	0%
	Assessing Context	0	0%
	Planning	0	0%
	Tailoring Strategies	0	0%
	Engaging Innovation Deliverers	0	0%
	Engaging Innovation Recipients	9	2.8%
	Doing	3	1.0%
	Reflecting and Evaluating	0	0%
	Adapting	1	0.3%
	Training	4	1.3%
	Integrating PrEP with other services	4	1.3%
	Other unspecified process	1	0.3%
	**Subtotal**	**23**	**7.3%**
**Total**		**316**	**100%**

#### Innovation characteristics

There were 43 measured determinants related to characteristics of PrEP, and most of these were *innovation* determinants (*n =* 33). Barriers impacting PrEP uptake and retention among patients related to concerns about PrEP side effects [54–57], costs of PrEP and co-pays associated with follow-up visits [57–60], and the inherent complexity of adhering to PrEP daily oral pill regiments as well as regular HIV testing [61]. Concerns were also raised regarding potential reduction in prescription medication effectiveness and other drug interactions [54]. Patient-reported facilitators to uptake and continued adherence included the fact that PrEP has been shown to be highly effective [22].

*“I didn’t take PrEP because I was worried about the stomach side effects…I’m already having a lot of stomach problems, so I didn’t want to put that on top of it. I was thinking about taking [PrEP], but then I didn’t want to risk getting sick….I probably should take [PrEP]…I just…the worry about side effects are outweighing the worry of getting AIDS”* [62].

Ten implementation determinants were assessed regarding PrEP characteristics. Barriers to implementation included the costs associated with providing PrEP care and related services [61]. Providers expressed concerns that influenced their decision to prescribe PrEP, such as potential side effects and safety, as well as drug interactions among individuals who use drugs and those with comorbid conditions [63]. Facilitators in this domain also included PrEP effectiveness and source of evidence [63]; that the existence of formal CDC guidelines supporting its use having the greatest influence over their decision to prescribe PrEP [64].

#### Outer setting

There were 47 *Outer Setting* determinants, with the majority being innovation-level (*n* = 27). Primary barriers at the *Outer Setting* included societal stigma [58, 61, 65], structural and systemic oppression [66], and perceived provider bias [66]. These barriers were deterrents for individuals with past negative healthcare experiences, resulting in their reluctance to engage with medical systems [66].

*The minute [the doctors] find out you are a drug addict, that you are an injection [drug] user, you can see it right in their face. They change their whole attitude. They do not want to help you…I hate telling the doctor that I use drugs…because they are going to blame anything wrong with you on the drug use”* [54].

Other key barriers included conditions such as not having stable housing or shelter [54, 63], not having reliable transportation to get refills or being present for regular follow-up visits [22], competing health priorities due to drug use and dependence [54], and lack of insurance to support financing [58]. Moreover, many PWID described that criminal justice involvement was a barrier that interrupted regular PrEP use [54]. PWID particularly mentioned that PrEP use could be interrupted by incarceration, which disconnects individuals from regular sources of medication, healthcare and social services [54]. Provider perspectives included barriers such as gentrification, wherein clients were being pushed out of urban centers where they could more easily access PrEP care [58]. *Outer Setting* facilitators to taking PrEP among recipients included perceptions that they were susceptible to HIV and thus needed PrEP based on the environment in which they lived [65]. For example, female sex workers reported risk and fear of sexual assault and reported that this motivated them to be on PrEP [65]. Finally, some participants reported that costs for entire PrEP regimens were fully covered by their insurance, which facilitated their adherence to PrEP [22].

*“Does insurance cover it? If there’s a copay or something on it, many people are not paying out of pocket for it. Obviously, I’m living on the street and don’t have a lot of money”* [61].

There were 20 *Outer Setting* implementation determinants reported in the literature. Key implementation barriers included healthcare systems not being adequately equipped to address the complex needs of individuals who use drugs [54], and policy-level requirements that PrEP be accessed through a medical provider [66]. Although some providers indicated that uncertainty around third-party reimbursement was a barrier to PrEP implementation [63], other providers, particularly in the South, noted that copayment assistance programs facilitated PrEP uptake among uninsured patients, and that financial barriers were not persistent to obtaining medication for most patients [56].

#### Inner setting

There were 23 determinants identified within the *Inner Setting.* Most were related to physical infrastructure, such as patient preference for where they prefer to receive PrEP. Often, patients indicated that they would prefer to receive PrEP in settings like the emergency room, drug treatment clinics, or through a syringe services program (SSP), rather than in an HIV clinic or primary care [65]. Another prevalent barrier included difficulties navigating the healthcare system in order to secure and retain access to PrEP [22]. Providers also recognized the importance of aligning PrEP delivery [62]. In accordance with patient preferences, suggesting that offering PrEP alongside methadone in a community drug treatment setting could facilitate monitoring of patient adherence to PrEP [61].

Inner setting *implementation* determinants included poor infrastructure for accurately assessing HIV risk and delivering PrEP, insufficient staffing, and limited provider capacity and/or willingness to prescribe and monitor adherence to PrEP, particularly for PWID [54].

*“…the infrastructure doesn’t really exist right now for [PrEP]” – CBO Program Manager* [54].

Primary care providers who were situated in a larger hospital setting that included substance use programs were associated with increased referral making compared to those that were not co-located [63]. Other inner-setting facilitators of PrEP implementation included having sufficient funding and resources for medication monitoring through blood tests [63], having relational connections with other programs to refer out to [63], and having a positive culture among providers who regarded themselves as having high levels of cultural competence shared professional ideals and values around providing care to populations disproportionately affected by HIV [66].

#### Individuals

The *Individuals* domain represented the majority of determinants (*n* = 180, 56%), and most of those were innovation determinants (*n* = 129, 41%).

#### Capability

There were 36 determinants related to individual capability, and of those, 28 were *innovation* determinants. Patient-level capability barriers for initiating and adhering to PrEP included difficulty remembering to consistently adhere to a daily dose of PrEP; individuals reported that the use of illicit drugs affected their cognitive ability to remember to adhere to the PrEP regimen [61, 62, 67], with missing a dose the prior day significantly predicting missing a dose on the next day [68]. PrEP knowledge (how to use PrEP) as well as general health literacy were also reported as common barriers [54, 66]. Perceived risk was low among individuals who were clinically identified as at risk for HIV [54, 57]. Low self-efficacy and a need to prioritize daily survival challenges also reportedly interfered with participants’ ability to take PrEP daily [62]. In an empirical model, behavioral skills were significantly predictive of PrEP use [15]. Facilitators related to capability included patient strategies such as putting their PrEP medication right next to their bed to prompt its use [61]. Moreover, individuals who reported accurate information about PrEP were associated with higher willingness to use PrEP [15].

Among the eight implementation barriers, provider-level capability barriers included limited knowledge of who PrEP is indicated for as well as it’s demonstrated safety and efficacy [63, 64]. Krakower et al. found that only 26% of nearly 1200 infectious disease physicians felt adequately prepared to prescribe PrEP to PWID [69]. This lack of knowledge also reportedly deterred providers from educating their clients about PrEP, when they themselves had limited information [63].

*“I don’t even know much about [PrEP]. If I don’t know much about it, how can I expect them [clients] to?” (Counselor)* [58].

#### Opportunity

There were 25 determinants related to individual opportunity, and all but one were *innovation* determinants. Patient-level opportunity barriers primarily included lack of awareness of PrEP, thus not having the opportunity to benefit from its use. Overall, lack of awareness that PrEP existed was one of the most pervasive individual-level barriers among both patients and providers. In their study, Shrestha et al. reported that 95% of their sample had never heard of PrEP [61]; Towe et al. reported that only 13% of their sample of 352 patients reported ever hearing about PrEP [57]. Kuo et al. also reported that only 13% of their sample had heard of PrEP [14]. In their sample of 400, Shrestha et al. reported that only 18% had heard of PrEP [70]; in their sample of 118 women who inject drugs, Walters et al. reported that 31% had heard of PrEP [71]. Similarly, few providers had heard of PrEP; Spector et al. reported that in their sample of 36, only four (11%) had heard of PrEP. Interestingly, women who reported transactional sex were over three times more likely to have heard about PrEP [71]. Relatedly, among women who inject drugs, those who had a conversation about HIV prevention with their provider were over seven times more likely to have heard of PrEP [71]. Another key opportunity barrier included perceived social consequences of taking PrEP, which included misperceptions that PrEP is associated with individuals who either already have HIV or use illicit drugs [22]. Providers also expressed that PrEP use might lead to intimate partner violence and retaliation [58]. On the other hand, some individuals reported that social influences of support from family members and friends facilitated their adherence to PrEP [22].

#### Motivation

This sub-category included 45 determinants with 39 being *innovation* determinants. One of the main motivational determinants of PrEP use among patients was perceived risk; those who acknowledged their vulnerability to HIV reported higher rates of willingness and intentions to take PrEP [15, 62, 65, 72]. However, the dynamic nature of perceived HIV risk impacted women who inject drugs (WWID) motivation to adhere to a daily PrEP regimen [62]. Many indicated fear of contracting HIV served as a significant driving force behind their interest and intentions to use PrEP [57, 65]. Regarding implementation determinants, some providers expressed concerns that PrEP use would impact risk compensation and lead to a reduction in condom use and needle sharing [61]; however, several studies highlighted provider enthusiasm and motivation to prescribe PrEP [61, 64, 73].

#### Other individual characteristics

This category included 52 determinants, with 41 being *innovation* determinants. These determinants captured differences in demographic variables such as gender, racial, or ethnic identity, sexual orientation, educational attainment, and different forms of drug use in relation to PrEP outcomes. Interestingly, drug use was found to act as both a facilitator for increased awareness and willingness to use PrEP [14, 74], as well as a barrier to PrEP adherence. Specifically, the use of club drugs (e.g., ketamine, ecstasy, GHB, cocaine, or methamphetamine) was associated with significantly higher odds of missing a PrEP dose on the day of drug use and the subsequent day [68] Additionally, individuals who used stimulants more frequently reported that their substance use would impact their ability to adhere to PrEP as prescribed [67].

Several studies have identified interesting patterns regarding PrEP initiation and retention among Hispanic/Latine individuals who use substances had higher PrEP initiation and retention [72, 75]. Educational attainment, however, yielded mixed results: one study showed that individuals with a college degree had lower odds of missing a PrEP dose [68], whereas, another found that lower educational attainment was associated with higher awareness of PrEP [74], and others found no association between educational attainment and PrEP awareness or uptake [71, 72]. One study revealed that individuals with a bisexual identity were more likely to be open to taking PrEP compared to those with a heterosexual identity [14]. No studies reported any association between gender and PrEP willingness or initiation [57, 59, 72]. Finally, cisgender women who had recently experienced physical sexual violence showed greater interest in PrEP than those who had not [76].

Implementation determinants in this category included findings such as providers over the age of 55 being less likely to be aware of PrEP compared to younger individuals [64]; and one study indicated that clinicians in larger practices (> 20 providers) were less inclined to prescribe PrEP than those in smaller practices [64].

#### Process determinants

A total of 23 process determinants were included. Common facilitative process determinants reported among recipients were the use of memory aids such as cell-phone reminders, post-it notes, therapist or provider-initiated reminders, and pill containers [22, 61]. Patients also identified social support networks, which facilitated adherence to PrEP [61], and they welcomed behavioral interventions to support PrEP adherence [61]. Embedding or integrating PrEP with existing services, such as within a methadone clinic or in a syringe exchange program were championed by both providers and patients [60, 61, 65]. Finally, being given a longer dose of medication beyond just a 7-day prescription was recommended [22]. Providers indicated that training and other educational supports were needed and welcomed to improve knowledge and awareness of PrEP to improve implementation [60, 63, 64]. To increase awareness of PrEP among patients and providers, mass media campaigns were recommended [58]. Finally, incentive programs for both patients and providers were recommended [58].

*“I think the best thing will be to give them with methadone because we are always going to make sure we get methadone. Because we will get sick if we don’t take it [methadone]. And as long as we can get it in the medication window, we’re not going to forget to take it”* [61].

### Change methods

Compared to the number of articles identifying determinants, there were very few studies evaluating change methods. In our search, we found two studies that utilized an implementation strategy to enhance the implementation of PrEP at the system/provider level. Additionally, we identified three adjunctive interventions that supported adoption and adherence to PrEP. Notably, three out of the four change methods focused on cisgender WWIDs. All four studies were feasibility and/or pilot trials. The core components and taxonomy coding for each of the change methods presented below are displayed in Table 3.

**Table 3:** Change method core components and classification

**Study Author**	**Implementation Strategy Name**	**Core Components**	**ERIC Domain**	**ERIC Strategy**
Bartholomew et al.	Incorporate PrEP into mobile SSP unit	Delivery of MOUD and PrEP at a mobile SSP	Change Infrastructure	Change Service Site
Roth et al.	Project SHE	Integrating PrEP within SSP	Change Infrastructure	Change Service Site
**Study Author**	**Adjunctive Intervention Name**	**Core Components**	**TDF Domain**	**COM-B**
Meyer et al.	Patient-centered PrEP decision aid	1. Discussion on the pros and cons of using PrEP related to other prevention strategies 2. Addressing domains identified as important to women with substance use disorders, including PrEP’s efficacy, cost, side effects, medication interactions, insurance coverage, and need for disclosure to partners.	Kn, Sk, BaCo, ECR	Psychological Capability, Reflective Motivation, Physical Opportunity
Richterman et al.	reSET-O	Mobile phone app that provides cognitive behavioral therapy (CBT) lessons, skill-building exercises, and contingency management in the form of small-value gift cards.	Kn, Sk, Re, BR	Psychological Capability, Automatic Reinforcement
Shrestha et al.	CHRP-BB	Group-based delivery across four 50-minute meetings. Sessions included the following topics: 1. Making the most of PrEP as an active health manager 2. Reducing drug risk and taking PrEP; 3. PrEP adherence and sex risk reduction strategies; 4. Negotiating partner support for HIV prevention	Kn, Sk, BaCa, BaCo, MADP, ECR, BR	Psychological Capability, Reflective Motivation, Physical Opportunity

Note: TDF abbreviations: Kn = Knowledge, Sk = Skills, BaCa = Beliefs about Capabilities, BaCo = Beliefs about Consequences,

Re = Reinforcement, MADP = Memory, Attention, and Decision Processes, ECR = Environmental Context and Resources,

SI = Social Influences, Em = Emotion, BR = Behavioral Regulation

### Implementation strategies

Roth et al., integrated PrEP care into a community-based syringe services program for cisgender WWID [77]. The study evaluated the program’s feasibility, acceptability, PrEP uptake, and retention at 12 and 24 weeks. Dissemination strategies included distributing flyers, program staff referrals, and informal peer recommendations. A total of 95 participants were enrolled, with 63 (66%) accepting a PrEP prescription in the first week. However, the number decreased to 48 at week 12 and 25 at week 24. These findings demonstrate the feasibility of integrating PrEP into existing services, which can enhance its reach and initial uptake. However, additional supports are necessary to improve patient retention over time.

Bartholomew et al. developed a strategy to incorporate PrEP into a mobile SSP unit in order to increase PrEP reach [78]. Authors conducted qualitative interviews with 30 Black PWIDs who were actively using SSP services to determine whether recipients perceived this strategy to be acceptable, appropriate, and feasible. Participants responded favorably to the strategy, indicating that it would address transportation, cost, and stigma barriers associated with accessing PrEP in a typical healthcare setting. Future research is needed that examines the effectiveness of and factors associated with implementing this strategy.

### Adjunctive interventions

Meyer et al. developed and piloted a decision aid for PrEP, which was implemented in an addiction treatment program [79]. During initial interviews, participants received a brief description of PrEP. Women who opted for ‘more information’ were engaged via the decision aid, which was created using REDCap and with the assistance of a trained research assistant. The purpose of evaluating the decision aid was to assess its impact on participants’ intentions to use PrEP. A total of 164 participants were enrolled, and 83 opted for more information and were engaged with the decision aid. The findings revealed that the decision aid significantly increased interest in PrEP from 25 to 89%, and it facilitated a transition along the PrEP care continuum [30] from interest to action, compared to the control group. The authors noted that individuals who perceived a higher risk of HIV and those with more severe alcohol use were more likely to opt for the decision aid. This tool could prove effective in preparing patients before meeting with clinicians, although further research with robust study designs is necessary to validate its use. Additionally, implementation considerations should be explored to determine the optimal delivery settings for this tool.

Richterman et al. assessed the acceptability of potentially adapting an existing mobile app, which supports opioid use disorder abstinence (reSET-O), to support PrEP uptake and adherence among women with opioid use disorder [80]. The authors interviewed 20 women at baseline regarding the app, finding that most thought an app would be acceptable, though several additional barriers such as inconsistent phone and internet access were identified. At 3-month follow-up, only two the participants had redeemed the prescription and downloaded the app, and both of those participants reported no recent opioid use. Five participants were interviewed at follow-up, none of whom downloaded the app. Reasons cited included technical difficulties, loss of phone, and not recalling being informed about the app. Incorporating a mobile-phone app as a means to support PrEP use among women with opioid use disorder revealed several implementation challenges and the need for additional implementation strategies. The core components of the app include providing cognitive behavioral therapy lessons, knowledge and skill-building exercises, and contingency management.

Shrestha et al. developed an integrated bio-behavioral approach to enhance PrEP adherence among PWUD [81]. The approach was tested with 40 participants recruited from a methadone maintenance program. The intervention, called the bio-behavioral community-friendly health recovery program (CHRP-BB) is based on behavior change theory and consists of four 50-minute group meetings. These meetings cover topics such as PrEP-specific information, risk perceptions, motivation to change, problem-solving skills to overcome barriers, weighing pros and cons, and improving decision-making related to PrEP while addressing stigma. The intervention also includes text message reminders sent to participants based on their preferred schedule to promote adherence to PrEP. The program’s feasibility and acceptability were assessed, along with self-reported PrEP adherence. Participants highly valued and accepted both program and the text message reminders. Self-reported PrEP adherence and knowledge significantly increased from baseline to the 1-month follow-up. Future randomized controlled trials are needed to demonstrate the comparative effectiveness of this program.

## Discussion

In this scoping review, we identified 32 articles that examined factors influencing implementation of PrEP by clinicians and its utilization by PWUD. We have categorized these factors using the updated CFIR2.0 framework, enabling us to analyze them across multiple levels and identify areas in need of additional research. Although most of the studies on determinants focused on PWIDs, a significant number were also found among individuals who use other modes of substance administration, such as ingesting, snorting, or smoking. However, this aggregation of injecting and non-injecting drug use poses a challenge, as previous research has shown distinct HIV risk behavior patterns between these two groups [82, 83]. Distinct implementation strategies to are needed to address barriers to HIV prevention between PWUD and PWID [84, 85]. For example, certain populations of individuals who use drugs, particularly cisgender MSM communities, have a greater propensity for methamphetamine use through injection [86]. Extensive research has shown that some cisgender MSM communities are more likely to engage in injecting practices due to the associated excitement, which is often fetishized and perceived to enhance intimacy between sexual partners [87]. This behavior is seen as facilitating or enhancing sexual experiences. Given these differences between injecting and non-injecting drug use, further research that distinguishes between these two groups is needed [85]. This differentiation is necessary to develop or tailor change methods that address the specific needs of different drug use methods among different populations.

Similar to the findings of a larger systematic review of PrEP [45], most determinants were found within the *Individuals* domain of CFIR. These determinants are linked to individual behavior change factors that impact the uptake and use by PrEP recipients. However, our findings also highlight key strategies for supporting the implementation and use of PrEP. Additionally, we identified five specific change methods for improving implementation and use that align closely with the identified determinants.

Although only two implementation strategies were identified, they both involved integrating PrEP into SSPs [77, 78]. Both patients and clinicians in our determinants review suggested embedding PrEP care within existing services, such as a methadone clinic or a syringe exchange program [22, 60, 61]. This innovative approach offers a convenient access point for PWUDs, reducing barriers and increasing reach. Additionally, strategies that alter the service site may facilitate clinician referral to such programs, as they can also provide adherence support and monitoring. Overcoming the barrier of prescribing PrEP to PWUD populations [15] becomes more feasible with this integrated approach. Moreover, SSPs have been identified as having the highest impact on preventing HIV among PWIDs [88]. Although these strategies have been identified as preferable to patients, to successfully implement, they also require additional implementation strategies to overcome the added need for additional organizational capacity resources such as staffing, training, time, and coordination. Further research is needed to explore the organizational-level determinants and to provide more evidence for how service integration into SSPs can be successfully implemented through the use of additional implementation strategies.

To address the consistently low awareness of PrEP revealed in numerous studies, additional implementation strategies are crucial. It is imperative to identify effective channels for informing and educating both providers and PWUD populations about PrEP. Merle et al.’s recent systematic review of change methods related to PrEP across various populations identified 18 implementation strategies [35]. Among these, ongoing clinician training to educate practitioners about the circumstances under which PrEP is indicated [31, 89] as well as clinical decision supports [32] have shown promise that can be adapted for use among PWUD.

To enhance engagement, uptake, and retention among recipients in PrEP programs, adjunctive interventions are essential alongside implementation support. PWUD face multilevel barriers such as housing instability [54], competing health concerns [63], challenges navigating the healthcare system [22], and individual levels barriers that impact capability, opportunity, and motivation to use PrEP. In this study, we identified three adjunctive interventions that sought to enhance adherence via education and decision aids [79], incorporating a behavioral approach to support motivation, problem-solving, and text message reminders [81], and via mobile app support [80]. These adjunctive interventions are crucial given the importance of adherence to PrEP, however, implementation considerations for how to support the adoption, fidelity, and sustainability are needed to ensure their use at scale [35]. Further, our review did not identify implementation strategies for structural interventions to address structural barriers (e.g., housing instability). A review of the CDC’s Prevention Research Synthesis Compendium of structural interventions for HIV identifies no structural interventions for PWID or PWUD that have been found to have an effect on PrEP [90]. However, structural interventions like the HRSA Homelessness Initiative have been found to increase viral suppression. Structural interventions like this should be examined for potential adaptations to be trialed for PrEP use, uptake, and adherence [91, 92]. Finally, such interventions will necessitate implementation strategies. Thus, such trials would benefit from hybrid implementation-effectiveness approaches to identify pre-implementation determinants, and identify strategies already embedded within settings that can be scaled up [93].

### Limitations and future directions

A number of factors should be considered when interpreting our findings. Our sampling methods reflect the scoping nature of our review; although it is likely that not every study meeting our inclusion criteria was captured, given that we did not search grey literature, we were able to provide guideposts for the direction of PrEP implementation research among PWUD. We also adapted CFIR and created several new codes (e.g., systemic oppression in the *Outer* Setting, individual characteristics not associated with behavior change in the *Individuals* domain, and multiple strategies to the *Process* domain). In addition, we expanded CFIR to encompass innovation determinants. Although CFIR was not designed to capture innovation determinants [94], we were unable to identify a framework that adequately captured recipient-level factors, which accounted for two-thirds of all determinants in our review due to the field’s focus on PrEP users rather than implementers and the delivery system. Regarding change method coding, we were limited in our ERIC and TDF/COM-B coding to what was available in the text. Future research on change methods should more carefully describe the methods used by specifying components in line with reporting recommendations [28]. In addition, we encourage researchers to utilize tools that link and test causal pathways between the change method components and their putative change mechanisms, determinants, and/or outcomes, such as the Implementation Research Logic Model (IRLM) [95]. Doing so will allow for more seamless data synthesis in future systematic reviews.

## Conclusion

To achieve the goals of *Ending the HIV Epidemic* (EHE) *Initiative*, a comprehensive approach is required, encompassing patient-, clinician-, and system-level determinants and developing, testing, and scaling-up effective adjunctive interventions and implementation strategies [34, 96]. Our results have important implications for clinical practice and public health initiatives. In addition to increasing access to PrEP in a timely manner, it is necessary to consider the unique contextual needs of PWUD when delivering PrEP services. We recommend exploring innovative models of service delivery that address cost and convenience issues. For instance, mobile units, compared to fixed site clinics, have been shown to be more accessible for marginalized populations [78]. We also suggest taking into account the importance of peer support and working with local communities to ensure a strong sense of trust and understanding among PWUD. Finally, we emphasize that PrEP uptake should be seen as an ongoing process rather than a one-time intervention, requiring clinicians to remain engaged with patients over time to ensure adherence to both PrEP itself and to the guidelines which require frequent HIV testing and other laboratory tests. Although adjunctive interventions play a crucial role in enhancing the intended outcomes, it is equally important to focus on factors that affect implementation; adjunctive interventions for PrEP should not only evaluate their impact on PrEP-related outcomes but also consider the unique implementation strategies required, which may differ from those used for PrEP alone. For example, implementing the community-friendly Health Recovery Program [81] will likely require resource allocation, infrastructure changes, provider support, facilitation, and other implementation strategies to ensure its sustained use in a given service setting. Our hope is that this study spurs on broader, larger sample studies developing and evaluating both implementation strategies and adjunctive interventions and bringing them to scale–with a prime focus on system and provider-level determinants to end the HIV epidemic.

### Electronic supplementary material

Below is the link to the electronic supplementary material.

Supplementary Material 1

## Data Availability

We included a supplemental file of our codebook, which includes operational definitions of codes. The systematic review dashboard, which a portion of our studies were drawn from is freely available at https://hivimpsci.northwestern.edu/dashboard/. Additional data is available from the authors upon request.

## Abbreviations

HIV: Human Immunodeficiency Virus
EHE: Ending the HIV Epidemic
PrEP: Pre–exposure Prophylaxis
PWUD: People who use drugs
PWID: People who inject drugs
WWID: Women who inject drugs
MSM: Men who have sex with men
CFIR: Consolidated Framework for Implementation Research
SSP: Syringe Services Program
COM: B–Capability, opportunity, motivation behavior change system
