# Supplementary material for: Pre-exposure prophylaxis (PrEP) among people who use drugs: a qualitative scoping review of implementation determinants and change methods
Source: Addict Sci Clin Pract. 2024 May 30;19:46. doi: 10.1186/s13722-024-00478-2 (PMC11138081; doi:10.1186/s13722-024-00478-2)
Supplement: Supplementary file 1 — Supplementary Material 1 [file 13722_2024_478_MOESM1_ESM.docx]

**Supplemental File 1**

***Code System for Determinants***

**1 Illustrative Quote**

Code illustrative quotes with this code to highlight in the paper

**2 Method of Drug Use**

**2.1 Method of Drug Use >> Both / Aggregated**

**2.2 Method of Drug Use >> Non-injecting**

**2.3 Method of Drug Use >> Intravenous / Injecting**

**3 Drug**

**3.1 Drug >> Unspecified**

**3.2 Drug >> Narcotic Analgesics**

Narcotic analgesics relieve pain, induce euphoria, and create mood changes in the user. Examples of narcotic analgesics include opium, codeine, heroin, demerol, darvon, morphine, methadone, Vicodin, and oxycontin.

**3.2.1 Drug >> Narcotic Analgesics >> darvon**

**3.2.2 Drug >> Narcotic Analgesics >> demerol**

**3.2.3 Drug >> Narcotic Analgesics >> oxycontin**

**3.2.4 Drug >> Narcotic Analgesics >> vicodin**

**3.2.5 Drug >> Narcotic Analgesics >> methodone**

**3.2.6 Drug >> Narcotic Analgesics >> morphine**

**3.2.7 Drug >> Narcotic Analgesics >> heroin**

**3.2.8 Drug >> Narcotic Analgesics >> codeine**

**3.2.9 Drug >> Narcotic Analgesics >> opium**

**3.2.10 Drug >> Narcotic Analgesics >> Suboxone**

**3.2.11 Drug >> Narcotic Analgesics >> Buprenorphine**

**3.3 Drug >> Cannabis**

Cannabis is the scientific name for marijuana. The active ingredient in cannabis is delta-9 tetrahydrocannabinol, or THC. This category includes cannabinoids and synthetics like Dronabinol.

**3.4 Drug >> Dissociative Anesthetics**

Dissociative anesthetics include drugs that inhibit pain by cutting off or dissociating the brain's perception of the pain.

**3.4.1 Drug >> Dissociative Anesthetics >> Ketamine**

**3.4.2 Drug >> Dissociative Anesthetics >> dextromethoraphan**

**3.4.3 Drug >> Dissociative Anesthetics >> PCP**

**3.4.4 Drug >> Dissociative Anesthetics >> other dissociative**

**3.5 Drug >> Depressant**

alcohol, barbiturates, anti-anxiety tranquilizers (e.g., Valium, Librium, Xanax, Prozac, and Thorazine), GHB (gamma hydroxybutyrate), Rohypnol, and many other anti-depressants (e.g., Zoloft, Paxil)

**3.5.1 Drug >> Depressant >> Alcohol**

**3.5.2 Drug >> Depressant >> G/GHB**

**3.5.3 Drug >> Depressant >> Heroin**

**3.5.4 Drug >> Depressant >> Other Depressant**

**3.6 Drug >> Halucinogens**

Hallucinogens cause the user to perceive things differently than they actually are

**3.6.1 Drug >> Halucinogens >> LSD**

**3.6.2 Drug >> Halucinogens >> Molly/MDMA/Ecstacy**

**3.6.3 Drug >> Halucinogens >> peyote**

**3.6.4 Drug >> Halucinogens >> psilocybin**

**3.6.5 Drug >> Halucinogens >> other halucinogen**

**3.7 Drug >> Inhalant**

Inhalants include a wide variety of breathable substances that produce mind-altering results and effects.

**3.7.1 Drug >> Inhalant >> Poppers**

**3.7.2 Drug >> Inhalant >> other inhalant**

Toluene, plastic cement, paint, gasoline, paint thinners, hair sprays, and various anesthetic gases.

**3.8 Drug >> Stimulants**

CNS stimulants accelerate the heart rate and elevate the blood pressure and "speed-up," or over-stimulate, the body

**3.8.1 Drug >> Stimulants >> Cocaine/Crack**

**3.8.2 Drug >> Stimulants >> Methamphetamine/Crank**

**3.8.3 Drug >> Stimulants >> Other Stimulant**

**4 Data Collection Method**

**4.1 Data Collection Method >> Quantitative**

**4.2 Data Collection Method >> Qualitative**

**4.3 Data Collection Method >> Mixed/Multi**

**5 Determinant Level**

**5.1 Determinant Level >> Innovation Determinant**

Determinant is at the patient / recipient level

**5.2 Determinant Level >> Implementation Determinant**

Determinant is at the delivery system / provider level

**6 Valence**

**6.1 Valence >> Facilitator**

**6.2 Valence >> Barrier**

**6.3 Valence >> Both**

**6.4 Valence >> Neither**

**6.5 Valence >> Unsure/Unspecified**

**7 Innovation Characteristics (Innovation Determinants)**

Innovation in this study is PrEP

**7.1 Innovation Characteristics (Innovation Determinants) >> 1.1.y: Innovation Source**

Innovation source: The degree to which the group that developed and/or visibly sponsored use of the innovation is reputable, credible, and/or trustable

- Mistrust of companies developing PrEP
- (Source refers to developer, not the broker [physician] trying to prescribe.)

**7.2 Innovation Characteristics (Innovation Determinants) >> 1.2.y: Evidence Base**

Evidence base: The degree to which the innovation (PrEP) has robust evidence support its efectiveness

- Concern that PrEP isn’t effective or valid
- Concern that PrEP isn’t necessary for a specific population

**7.3 Innovation Characteristics (Innovation Determinants) >> 1.3.y: Relative Advantage**

- **Relative advantage** of oral PrEP vs injectable PrEP or other forms of PrEP
- Patient preference

**7.4 Innovation Characteristics (Innovation Determinants) >> 1.4.y: Adaptability**

Adaptability: The degree to which the innovation can be modified, tailored, or refined to fit local context or needs

**7.5 Innovation Characteristics (Innovation Determinants) >> 1.5.y: Trialability**

Trialability: The degree to which the innovation can be tested or piloted on a small scale and undone

**7.6 Innovation Characteristics (Innovation Determinants) >> 1.6.y: Complexity**

Complexity: The degree to which the innovation is complicated, which may be reflected by its scope and/or the nature and number of connections and steps

- Pts’ thoughts on ease/difficulty
- Accessing through health system is difficult

**7.7 Innovation Characteristics (Innovation Determinants) >> 1.7.y: Design**

- **Design** (e.g., oral vs injectable), including PrEP messaging campaigns (1.7.y)

**7.8 Innovation Characteristics (Innovation Determinants) >> 1.8.y: Cost**

- **Cost**, including cost to obtain (1.8.y)
- Pts’ concerns about out-of-pocket costs (co-pays, associated labs)

**7.9 Innovation Characteristics (Innovation Determinants) >> 1.9.y: Other Innovation Characteristic**

- **Other innovation characteristic** that does not fit above (1.9.y)
- Concerns about side effects and risk compensation
- Downstream/surrounding benefits of PrEP (e.g., it is empowering, reduced HIV anxiety)

**8 Innovation Characteristics (Implementation Determinants)**

The innovationf or this study is PrEP

**8.1 Innovation Characteristics (Implementation Determinants) >> 1.1.x: Innovation Source**

Innovation Source: The group that developed and/or visibly sponsored use of the innovation is reputable, credible, and/or trustable.

**8.2 Innovation Characteristics (Implementation Determinants) >> 1.2.x: Evidence-Base**

· **Evidence base** (1.2.x)

- Concerns about effectiveness and safety in different populations

**8.3 Innovation Characteristics (Implementation Determinants) >> 1.3.x: Relative Advantage**

- **Relative advantage** of oral vs injectable or other forms of PrEP
- Provider preferences

**8.4 Innovation Characteristics (Implementation Determinants) >> 1.4.x: Adaptability**

Adaptability: The degree to which the innovation can be modified, tailored, or refined to fit local context or needs

**8.5 Innovation Characteristics (Implementation Determinants) >> 1.5.x: Trialability**

Trialability: The degree to which the innovation can be tested or piloted on a small scale and undone

**8.6 Innovation Characteristics (Implementation Determinants) >> 1.6.x: Complexity**

Complexity: The degree to which the innovation is complicated, which may be reflected by its scope and/or the nature and number of connections and steps

- Too complex for provider to deliver

**8.7 Innovation Characteristics (Implementation Determinants) >> 1.7.x: Design**

Design (e.g., oral vs injectable), including PrEP messaging campaigns

**8.8 Innovation Characteristics (Implementation Determinants) >> 1.8.x: Cost**

- **Cost**, including cost to deliver (1.8.x)
- Cost to pt is too high, so won’t be able to deliver
- Cost of using specific test is too high for deliverer
- Injectable is not cost-effective/savings

**8.9 Innovation Characteristics (Implementation Determinants) >> 1.9.x: Other Innovation Characteristics**

Other innovation characteristics: Other characteristics of the innovation that do not fit above

**9 Outer Setting (Innovation Determinants)**

**9.1 Outer Setting (Innovation Determinants) >> 2.1.y: Critical Incidents**

**Critical incidents:** Large-scale and/or unanticipated events disrupt the Outer Setting during implementation and/or delivery of the innovation (2.1.y)

o Ex: Patient cannot access PrEP due to quarantine or due to public transit stoppage

**9.2 Outer Setting (Innovation Determinants) >> 2.2.y: Local Attitudes**

o **Local Attitudes:** Sociocultural values (e.g., shared responsibility in public health) and beliefs (e.g., convictions about who needs and deserves PrEP) encourage the Outer Setting to support receipt of the innovation. (2.2.y)

Includes stigma

**9.3 Outer Setting (Innovation Determinants) >> 2.3.y: Local Conditions**

**Local Conditions:** Economic, environmental, political, and/or technological conditions enable the Outer Setting to support implementation and/or delivery of the innovation. (2.3.y)

**9.4 Outer Setting (Innovation Determinants) >> 2.4.y: Partnerships & Connections**

**Partnerships & connections** (2.4.y)

- Social endorsements or discouragement
- Social support in obtaining PrEP

**9.5 Outer Setting (Innovation Determinants) >> 2.5.y External Pressure**

· **External Pressure** (2.5.y)

o Other external forces of pressure

**9.5.1 Outer Setting (Innovation Determinants) >> 2.5.y External Pressure >> 2.5b.y. Societal Pressure**

o **Societal Pressure** (2.5b.y) Mass media campaigns, advocacy groups, social movements or protests drive need or desire to uptake PrEP

**9.6 Outer Setting (Innovation Determinants) >> 2.6.y: Policies & Laws**

**Policies & Laws** (2.6.y)

o CDC guidelines, FDA approval, WHO recommendations that encourage or prevent uptake

**9.7 Outer Setting (Innovation Determinants) >> 2.7.y: Financing**

F**inancing** (2.6b.y)

- Insurance companies will cover costs for patients
- If uninsured, government will cover
  1. **Outer Setting (Innovation Determinants) >> 2.8.y: Structural/Systemic Oppression**

o Identification of structural/systemic racism, cissexism (i.e., anti-transgender oppression), sexism, ableism, etc. as determinant hindering equitable uptake of or access to PrEP

- Structural: the totality of societal structures and policies that create and maintain inequities by unequally distributing access to opportunities and societal resources Systemic: embedded in the foundations of a society

**10 Outer Setting (Implementation Determinants)**

**10.1 Outer Setting (Implementation Determinants) >> 2.1.x: Critical Incidents**

Critical incidents: Large-scale and/or unanticipated events disrupt the Outer Setting during implementation and/or delivery of the innovation (2.1.x)

o Ex: shortage of PrEP due to COVID, natural disaster, etc.

**10.2 Outer Setting (Implementation Determinants) >> 2.2.x: Local Attitudes**

· **Local Attitudes:** Sociocultural values (e.g., shared responsibility in helping recipients) and beliefs (e.g., convictions about the worthiness of recipients) encourage the Outer Setting to support implementation and/or delivery of the innovation. (2.2.x)

Includes Stigma

**10.3 Outer Setting (Implementation Determinants) >> 2.3.x: Local Conditions**

**Local Conditions:** Economic, environmental, political, and/or technological conditions enable the Outer Setting to support implementation and/or delivery of the innovation. (2.3.x)

**10.4 Outer Setting (Implementation Determinants) >> 2.4.x: Partnerships & Connections**

**Partnerships & connections** (2.4.x)

o Inner Setting is networked with external entities, including referral networks, academic affiliations, and professional orgs. that enable implementation

**10.5 Outer Setting (Implementation Determinants) >> 2.5.x: External Pressure**

External Pressure: o Other external forces of pressure other than market or societal pressure

**10.5.1 Outer Setting (Implementation Determinants) >> 2.5.x: External Pressure >> 2.5a.x: Market Pressure**

Market Pressure (2.5a.x)

o Not enough providers around, so we will move into that space

o Others are doing it, so we need to as well to be competitive

**10.5.2 Outer Setting (Implementation Determinants) >> 2.5.x: External Pressure >> 2.5b.x: Societal Pressure**

Societal Pressure (2.5b.x)

o Mass media campaigns, advocacy groups, social movements or protests drive need to implement and deliver the innovation

**10.5.3 Outer Setting (Implementation Determinants) >> 2.5.x: External Pressure >> 2.5d.x: Performance-Measurement Pressure**

Performance-measurement pressure: Quality or benchmarking metrics and externally established service goals

o Meeting their PrEP quotas set by funders

**10.6 Outer Setting (Implementation Determinants) >> 2.6.x: Policies & Laws**

**Policies & Laws** (2.6.x)

o CDC guidelines, FDA approval, WHO recommendations

o Expert disagreement/uncertainty around who the best targets are

**10.7 Outer Setting (Implementation Determinants) >> 2.7.x: Financing**

Financing: Funding provided by third-party or service reimbursements (e.g., foundations, government, insurance companies)

o Insurance companies will reimburse the cost

o Public health department should take on cost burden

**10.8 Outer Setting (Implementation Determinants) >> 2.8.x: Structural/Systemic Oppression**

o Identification of structural/systemic racism, cissexism (i.e., anti-transgender oppression), sexism, ableism, etc. as determinant hindering equitable delivery of PrEP

o Structural: the totality of societal structures and policies that create and maintain inequities by unequally distributing access to opportunities and societal resources Systemic: embedded in the foundations of a society

**11 Inner Setting**

**11.1 Inner Setting >> 3.1: Structural Characteristics**

· **Structural characteristics** (3.1) – Other structural characteristics not included in physical infrastructure, information technology infrastructure, or work infrastructure

**11.1.1 Inner Setting >> 3.1: Structural Characteristics >> 3.1a: Physical Infrastructure**

Physical Infrastructure (3.1a) - Layout and configuration of space and other tangible material features supports implementation and/or delivery of the innovation.

**11.1.2 Inner Setting >> 3.1: Structural Characteristics >> 3.1b: Information Technology Infrastructure**

Information Technology Infrastructure (3.1b) - Technological systems for tele-communication, electronic documentation, and data storage, management, reporting, and analysis supports implementation and/or delivery of the innovation

**11.1.3 Inner Setting >> 3.1: Structural Characteristics >> 3.1c: Work Infrastructure**

Work Infrastructure (3.1c) - Organization of tasks and responsibilities, within and between individuals and teams, supports implementation and/or delivery of the innovation

**11.2 Inner Setting >> 3.2a: Relational Connections**

Relational characteristics: Formal and informal relationships, networks, and interactions within and across structural, professional, or other Inner Setting boundaries

**11.3 Inner Setting >> 3.2b: Communication**

Communications: Formal and informal information sharing

**11.4 Inner Setting >> 3.3: Culture**

Culture: other aspects of culture other than equity-/recipient-/deliverer-/learning- centeredness

**11.4.1 Inner Setting >> 3.3: Culture >> 3.3a: Equity-Centeredness**

Equity-Centeredness (3.3a) – shared values, beliefs, and norms surrounding equity within the implementation site

**11.4.2 Inner Setting >> 3.3: Culture >> 3.3b: Recipient-Centeredness**

Recipient-Centeredness (3.3b) - shared values, beliefs, and norms around caring, supporting, and addressing the needs and welfare of recipients

**11.4.3 Inner Setting >> 3.3: Culture >> 3.3c: Deliverer-Centeredness**

Deliverer-Centeredness (3.3c) - shared values, beliefs, and norms around caring, supporting, and addressing the needs and welfare of deliverers

**11.4.4 Inner Setting >> 3.3: Culture >> 3.3d: Learning-Centeredness**

Learning-Centeredness (3.3d) - shared values, beliefs, and norms around psychological safety, continual improvement, and using data to inform practice

**11.5 Inner Setting >> 3.4: Tension for Change**

Tension for change: The degree to which the current situation is intolerable and needs to change

**11.6 Inner Setting >> 3.5: Compatibility**

Compatibility: The degree to which the innovation fits with workflows, systems, and processes

- Statements about how settings (e.g., pharmacies) already have practices conducive to delivering PrEP – purview paradox

**11.7 Inner Setting >> 3.6: Relative Priority**

Relative priority: The degree to which implementing and delivering the innovation is important compared to other initiatives

**11.8 Inner Setting >> 3.7: Incentive Systems**

Incentive systems: (3.7) - Tangible and/or intangible incentives and rewards and/or disincentives and punishments support implementation and delivery of the innovation.

**11.9 Inner Setting >> 3.8: Mission Alignment**

Mission Alignment: The degree to which implementing and delivering the innovation is in line with the overarching commitment, purpose, or goals of the Inner Setting

- Statements about howPrEP aligns with the goals of the setting/organization

**11.10 Inner Setting >> 3.9: Available Resources**

Available resources: Resources other than funding, space, materials/equipment

**11.10.1 Inner Setting >> 3.9: Available Resources >> 3.9a: Funding**

Funding (3.9a) – Funding is available to implement and deliver the innovation

**11.10.2 Inner Setting >> 3.9: Available Resources >> 3.9b: Space**

Space (3.9b) – Physical space is available to implement and deliver the innovation

**11.10.3 Inner Setting >> 3.9: Available Resources >> 3.9c: Materials & Equipment**

Materials & Equipment (3.9c) – Supplies are available to implement and deliver the innovation

**11.10.4 Inner Setting >> 3.9: Available Resources >> 3.9d: Access to Knowledge & Information**

Access to Knowledge & Information (3.9d) – Guidance and/or training is accessible to implement and deliver the innovation

**11.10.5 Inner Setting >> 3.9: Available Resources >> 3.9e: Staffing**

Staffing is available to implement and deliver the innovation.

E.g.: staffing is sufficient/insufficient for successful implementation of the innovation; not enough staff; high turnover; not enough financial resources to hire more staff; too many staff in some positions and not enough in others

**12 Characteristics of Individuals (Innovation Determinants)**

**12.1.2 Characteristics of Individuals (Innovation Determinants) >> 13.1.1h.y: Innovation Deliverers >> 12.1.1h.c.y Opportunity**

Perception of the degree to which they have availability, scope, and power to fulfill their role in the implementation of PrEP.

Includes social opportunity - such as social influences and approval from others

and physical opportunity, such environmental context and resources (i.e., how their physical or social environment is set up

**12.1.3 Characteristics of Individuals (Innovation Determinants) >> 13.1.1h.y: Innovation Deliverers >> 12.1.1h.d.y Motivation**

Perception of the degree to which they are committed to fulfilling their role in the implementation of PrEP

Includes automatic motivation: reinforcement (tangible incentives that increase / decrease behavior, social reward); or emotional responses.

and Reflective Motivation (beliefs about one's capabilities, beliefs about consequences of enacting the behavior, an individual's social or professional role or identify, having goals or intentions related to enacting the beahvior, optimism

**12.1.4 Characteristics of Individuals (Innovation Determinants) >> 13.1.1h.y: Innovation Deliverers >> 12.1.1h.e.y: Characteristics Not Associated with Behavior**

Individual level determinant that is not related to their behavior, but more just their demographic category.

**12.2 Characteristics of Individuals (Innovation Determinants) >> 13.1.1i.y: Innovation Recipients**

Don't Code

**12.2.1 Characteristics of Individuals (Innovation Determinants) >> 13.1.1i.y: Innovation Recipients >> 12.2.1i.b.y Capability/Self-Efficacy**

Perception of the degree to which the recipient has interpersonal competence, knowledge, and skills to access PrEP, when to get tested, where to find care after PrEP, what to do after diagnosis, etc.

Includes psychological capability (memory, attention, decision process, ability to regulate own behavior, cognitive and interpersonal skills as well as physical capability, such as how to enact the behavior needed to adhere to the intervention.

**12.2.2 Characteristics of Individuals (Innovation Determinants) >> 13.1.1i.y: Innovation Recipients >> 12.2.1i.c.y Opportunity**

Perception of the degree to which the recipient has availability, scope, and power to access PrEP.

Includes social opportunity - such as social influences and approval from others

and physical opportunity, such environmental context and resources (i.e., how their physical or social environment is set up

**12.2.3 Characteristics of Individuals (Innovation Determinants) >> 13.1.1i.y: Innovation Recipients >> 12.2.1i.d.y Motivation**

Perception of the degree to which the recipient is committed to PrEP

Includes automatic motivation: reinforcement (tangible incentives that increase / decrease behavior, social reward); or emotional responses.

and Reflective Motivation (beliefs about one's capabilities, beliefs about consequences of enacting the behavior, an individual's social or professional role or identify, having goals or intentions related to enacting the beahvior, optimism

**12.2.4 Characteristics of Individuals (Innovation Determinants) >> 13.1.1i.y: Innovation Recipients >> 12.2.i.e.y: Characteristics Not Associated with Behavior**

Individual level determinant that is not related to their behavior, but more just their demographic category.

**13 Characteristics of Individuals (Implementation Determinants)**

**13.1 Characteristics of Individuals (Implementation Determinants) >> 13.1.1h.x: Innovation Deliverers**

providers

**13.1.1 Characteristics of Individuals (Implementation Determinants) >> 13.1.1h.x: Innovation Deliverers >> 13.1.1h.b.x Capability/Self-Efficacy**

Perception of the degree to which the they have interpersonal competence, knowledge, and skills to fulfill their role in the implementation of PrEP.

Includes psychological capability (memory, attention, decision process, ability to regulate own behavior, cognitive and interpersonal skills as well as physical capability, such as how to enact the behavior needed to adhere to the intervention.

**13.1.2 Characteristics of Individuals (Implementation Determinants) >> 13.1.1h.x: Innovation Deliverers >> 13.1.1h.c.x Opportunity**

Perception of the degree to which they have availability, scope, and power to fulfill their role in the implementation of PrEP.

Includes social opportunity - such as social influences and approval from others

and physical opportunity, such environmental context and resources (i.e., how their physical or social environment is set up

**13.1.3 Characteristics of Individuals (Implementation Determinants) >> 13.1.1h.x: Innovation Deliverers >> 13.1.1h.d.x Motivation**

Perception of the degree to which they are committed to fulfilling their role in the implementation of PrEP

Includes automatic motivation: reinforcement (tangible incentives that increase / decrease behavior, social reward); or emotional responses.

and Reflective Motivation (beliefs about one's capabilities, beliefs about consequences of enacting the behavior, an individual's social or professional role or identify, having goals or intentions related to enacting the beahvior, optimism

**13.1.4 Characteristics of Individuals (Implementation Determinants) >> 13.1.1h.x: Innovation Deliverers >> 13.1.1h.e.x: Characteristics Not Associated with Behavior**

Individual level determinant that is not related to their behavior, but more just their demographic category.

**13.2 Characteristics of Individuals (Implementation Determinants) >> 13.1.1i.x: Innovation Recipients**

**13.2.1 Characteristics of Individuals (Implementation Determinants) >> 13.1.1i.x: Innovation Recipients >> 13.1.1i.b.x Capability/Self-Efficacy**

Perception of the degree to which the they have interpersonal competence, knowledge, and skills to fulfill their role in the implementation of PrEP.

Includes psychological capability (memory, attention, decision process, ability to regulate own behavior, cognitive and interpersonal skills as well as physical capability, such as how to enact the behavior needed to adhere to the intervention.

**13.2.2 Characteristics of Individuals (Implementation Determinants) >> 13.1.1i.x: Innovation Recipients >> 13.1.1i.c.x Opportunity**

Perception of the degree to which they have availability, scope, and power to fulfill their role in the implementation of PrEP.

Includes social opportunity - such as social influences and approval from others

and physical opportunity, such environmental context and resources (i.e., how their physical or social environment is set up

**13.2.3 Characteristics of Individuals (Implementation Determinants) >> 13.1.1i.x: Innovation Recipients >> 13.1.1i.d.x Motivation**

Perception of the degree to which the recipient is committed to PrEP

Includes automatic motivation: reinforcement (tangible incentives that increase / decrease behavior, social reward); or emotional responses.

and Reflective Motivation (beliefs about one's capabilities, beliefs about consequences of enacting the behavior, an individual's social or professional role or identify, having goals or intentions related to enacting the beahvior, optimism

**13.2.4 Characteristics of Individuals (Implementation Determinants) >> 13.1.1i.x: Innovation Recipients >> 13.1.1i.e.x: Characteristics Not Associated with Behavior**

Individual level determinant that is not related to their behavior, but more just their demographic category.

**14 Process**

**14.1 Process >> 5.1: Teaming**

**Teaming** (5.1) – Perceptions of the degree to which individuals join together, intentionally coordinating and collaborating on interdependent tasks, to implement the innovation

**14.2 Process >> 5.2: Assessing for Needs**

Assessing for needs (5.2) – Other themes related to assessing needs that aren’t related to sub-codes

**14.2.1 Process >> 5.2: Assessing for Needs >> 5.2a: Innovation Deliverers**

Innovation Deliverers (5.2a) – Perceptions of the degree to which individuals collect information about the priorities, preferences, and needs of deliverers to guide implementation and delivery of the innovation

**14.2.2 Process >> 5.2: Assessing for Needs >> 5.2b: Innovation Recipients**

Innovation Recipients (5.2b) – Perceptions of the degree to which individuals collect information about the priorities, preferences, and needs of recipients to guide implementation and delivery of the innovation

**14.3 Process >> 5.3: Assessing Context**

Assessing Context (5.3) – Perceptions of the degree to which individuals collect information to identify and appraise barriers and facilitators to implementation and delivery of the innovation

**14.4 Process >> 5.4: Planning**

- **Planning** (5.4) – Perceptions of the degree to which individuals identify roles and responsibilities, outline specific steps and milestones, and define goals and measures for implementation success in advance

**14.5 Process >> 5.5: Tailoring Strategies**

Tailoring Strategies (5.5) – Perceptions of the degree to which individuals choose and operationalize implementation strategies to address barriers, leverage facilitators, and fit context

**14.6 Process >> 5.6: Engaging**

- **Engaging** (5.6) – Other themes related to engaging that are not included in subcodes

**14.6.1 Process >> 5.6: Engaging >> 5.6a: Innovation Deliverers**

Innovation Deliverers (5.6a) – Perceptions of the degree to which individuals attract and encourage deliverers to serve on the implementation team and/or to deliver the innovation

**14.6.2 Process >> 5.6: Engaging >> 5.6b: Innovation Recipients**

Innovation Recipients (5.6b) -- Perceptions of the degree to which individuals attract and encourage recipients to serve on the implementation team and/or participate in the innovation

**14.7 Process >> 5.7: Doing**

Doing: Perceptions of the degree to which individuals implement in small steps, tests, or cycles of change to trial and cumulatively optimize delivery of the innovation

**14.8 Process >> 5.8: Reflecting and Evaluating**

Reflecting and evaluating: Other themes related to reflecting & evaluating other than those included in subcodes

**14.8.1 Process >> 5.8: Reflecting and Evaluating >> 5.8a: Implementation**

Perceptions of the degree to which individuals collect and discuss quantitative and qualitive information that indicates the degree to which implementation outcomes are achieved

**14.8.2 Process >> 5.8: Reflecting and Evaluating >> 5.8b: Innovation**

Innovation (5.8b) – Perceptions of the degree to which individuals collect and discuss quantitative and qualitive information that indicates the degree to which innovation outcomes are achieved

**14.9 Process >> 5.9: Adapting**

**Adapting** (5.9) – Perceptions of the degree to which individuals modify the innovation and/or the Inner Setting for optimal fit and integration into delivery routines

**14.10 Process >> 5.10 Training**

The extent to which providers indicate that training is needed to succesfully implement the innovation

**14.11 Process >> 5.11 Integrating PrEP**

Integrating PrEP into an existing service setting would improve an implementation outcome
